# Inhibiting MDSC differentiation from bone marrow with phytochemical polyacetylenes drastically impairs tumor metastasis

**DOI:** 10.1038/srep36663

**Published:** 2016-11-18

**Authors:** Wen-Chi Wei, Sheng-Yen Lin, Chun-Wen Lan, Yu-Chen Huang, Chih-Yu Lin, Pei-Wen Hsiao, Yet-Ran Chen, Wen-Chin Yang, Ning-Sun Yang

**Affiliations:** 1 Agricultural Biotechnology Research Center, Academia Sinica, ROC, Taiwan; 2 Graduate Institute of Life Sciences, National Defense Medical Center, Taipei, ROC, Taiwan; 3 Institute of Biomedical Sciences, National Sun Yat-Sen University, Kaohsiung, ROC, Taiwan

## Abstract

Myeloid-derived suppressor cells (MDSCs) are implicated in the promotion of tumor metastasis by protecting metastatic cancerous cells from immune surveillance and have thus been suggested as novel targets for cancer therapy. We demonstrate here that oral feeding with polyacetylenic glycosides (BP-E-F1) from the medicinal plant *Bidens pilosa* effectively suppresses tumor metastasis and inhibits tumor-induced accumulation of granulocytic (g) MDSCs, but does not result in body weight loss in a mouse mammary tumor-resection model. BP-E-F1 is further demonstrated to exert its anti-metastasis activity through inhibiting the differentiation and function of gMDSCs. Pharmacokinetic and mechanistic studies reveal that BP-E-F1 suppresses the differentiation of gMDSCs via the inhibition of a tumor-derived, G-CSF-induced signaling pathway in bone marrow cells of test mice. Taken together, our findings suggest that specific plant polyacetylenic glycosides that target gMDSC differentiation by communicating with bone marrow cells may hence be seriously considered for potential application as botanical drugs against metastatic cancers.

Owing to the recent advancement in precision surgeries, early diagnosis of cancer, and adjuvant therapies used alongside chemotherapeutic drugs, cancer death rate now mainly reflects the degree and pattern of residual or circulating tumor cells metastasizing from the primary tumor site to secondary tissue target sites^1^,^2^,^3^. Therefore, control, blockage and prevention of such metastasis have been recognized as key steps for successful intervention^4^,^5^,^6^. Cancer cells can migrate from the primary tumor site to various specific tissues/organs in the body via the bloodstream, the lymphatic system or other routes^7^,^8^. To facilitate successful migration, tumor cells have been suggested to render selected target organ microenvironments permissive to implantation and outgrowth of circulating tumor cells. This can be achieved by inducing stromal cells and other immunosuppressive cells to secrete specific cytokines or growth factors. The tissue microenvironments manipulated in this way by tumor cells have been termed pre-metastatic niches^9^,^10^. Therefore, tumor-induced immunosuppression activity is a key element that enables tumors to escape immune surveillance, enabling growth at both primary and metastatic sites^11^,^12^,^13^.

Myeloid-derived suppressor cells (MDSCs) and T-regulatory cells (Tregs) are the two main immunosuppressive cell types that negatively regulate immune responses against cancers^14^,^15^,^16^,^17^. These cells have been found largely responsible for inhibiting host antitumor immunities and consequently impairing the effectiveness of anticancer immunotherapeutic approaches^18^. MDSCs are a heterogeneous population of cells that consist of myeloid progenitor cells and immature myeloid cells (IMCs) that are present during tumor progression, tissue inflammation and pathogen infection^16^,^19^. Two different subtypes of MDSCs, monocytic MDSCs and granulocytic MDSCs (mMDSCs and gMDSCs), have been identified^20^,^21^,^22^ and the expansion and activation of MDSCs have been shown to be triggered by a number of tumor- or tumor stromal cell-derived factors^23^,^24^,^25^. For instance, tumor-derived granulocyte-colony-stimulating factor (G-CSF) has been identified as a major factor for the differentiation of gMDSCs, and tumor-derived granulocyte-macrophage colony-stimulating factor (GM-CSF) has been shown to play a key role in mMDSC production^26^,^27^,^28^,^29^. The immature MDSCs infiltrate into a specific tumor microenvironment and can differentiate into tumor-associated macrophages (TAM)^30^. Various MDSCs have therefore been recognized to play a hierarchical role in tumor-induced immunosuppression activity. As a result, preventing the development of MDSCs is being considered as a promising strategy for fighting cancerous diseases. Our current study was designed to investigate this strategy against mammary tumor metastasis in a clinically relevant mouse model, by using a specific phytochemical group as the modifiers of gMDSC activities.

*Bidens pilosa* L., an Asteraceae plant, has been traditionally used as a folk medicine and an herbal tea constituent in many Asian cultures. Plant extracts of *B. pilosa* have been reported to confer a spectrum of immunomodulatory and anti-inflammatory activities. Previously, we reported that a phytoextract and phytochemicals isolated from *B. pilosa* exhibit immunomodulatory activities that regulate the functions of dendritic cells and T cells^31^,^32^,^33^. In the present study, we showed that high levels of expression of G-CSF activity and gMDSC populations are readily detected in a murine 4T1 mammary carcinoma model. An ethanol extract of *B. pilosa* (BP-E) was found to exhibit a strong immunomodulatory effect that effectively suppressed the G-CSF-induced differentiation of gMDSCs from test bone marrow cells *ex vivo*. *In vivo* studies further demonstrated that simple oral delivery of BP-E can suppress 4T1 tumor metastasis with high potency in a tumor-resection model. Mechanistically, we show that this effect of BP-E is mediated via inhibiting the differentiation and function of gMDSCs in test mice. Bio-organic chemical analysis showed that a specific group of polyacetylenic glycosides, which made up the great majority (>89%) of the constituents of one fraction of BP-E (BP-E-F1), act as the active phytochemicals responsible for the detected MDSC activities *ex vivo* and *in vivo*, and the resultant anti-metastatic activities *in vivo*. Taken together, our results strongly suggest that specific polyacetylene phytochemicals in BP plant extracts and the derived ethanol fraction may have therapeutic or other clinical applications against metastasis of mammary tumor. Therefore, we would like to consider that gMDSC activities/populations have potential to serve as an immune checkpoint modifier. Our findings on BP-E-F1 phytochemicals may also have applications as botanical drugs against metastatic cancers.

## Results

### Change in myeloid derived suppressor cell populations and G-CSF level in blood and spleen tissues of murine 4T1 tumor-bearing mice

**Figure 1:**
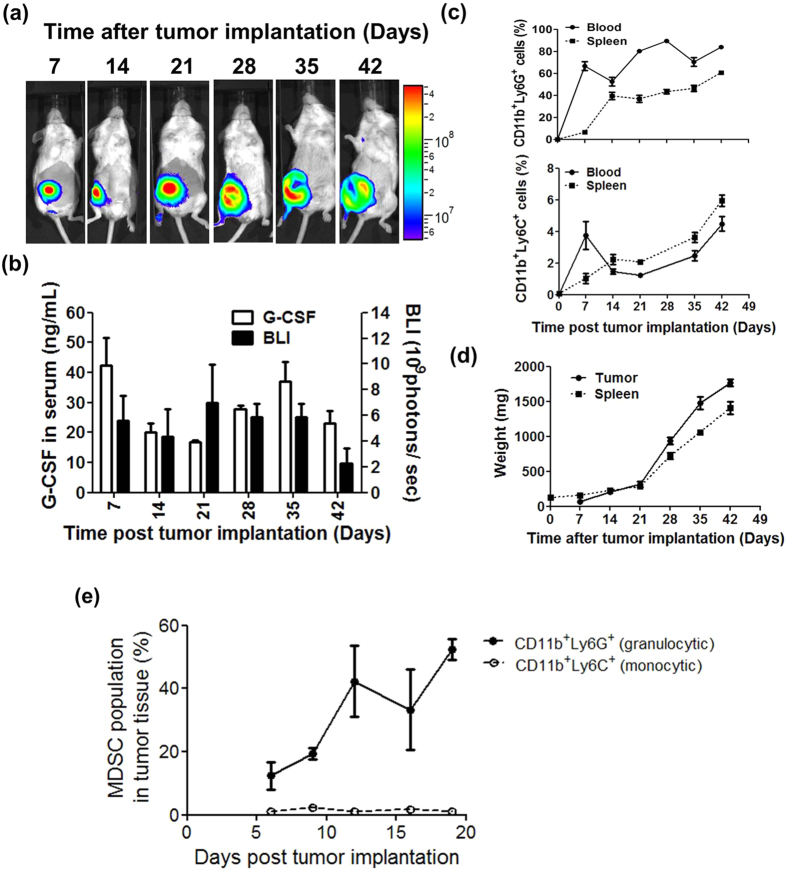
Change in myeloid-derived suppressor cell populations and G-CSF level in blood and spleen tissues of murine 4T1 tumor-bearing mice. Test mice were implanted orthotopically with 5 × 10^5^ 4T1-luc2 cells and monitored weekly by non-invasive bioluminescence imaging (BLI). (**a**) Representative weekly BLI of tumor-bearing mice. (**b**) Quantitation of BLI of test tumors (**a**) (black bar) and expression of serum G-CSF level (white bar) in tumor-bearing mice. (**c**) Population distribution of granulocytic myeloid derived suppressor cells (gMDSCs) and monocytic myeloid derived suppressor cells (mMDSCs) in blood cells (dark line) and splenocytes (dotted line) in tumor-bearing mice, analyzed by flow cytometry. (**d**) Weight of tumor mass (dark line) and spleen (dotted line) in tumor-bearing mice. (**e**) Population of gMDSCs (solid line/close circle) and mMDSCs (dotted line/open circle) in tumor tissues were determined by flow cytometry analysis.

The MDSC population has been shown to increase in cancer patients^34^,^35^,^36^. Relatedly, granulocyte colony-stimulating factor (G-CSF) was shown to be one of the key cytokines secreted by tumor cells to mediate MDSC production^27^. In order to characterize the dynamic change in the MDSC population and G-CSF expression in 4T1 tumor-bearing mice, transgenic 4T1-luc2 cells were orthotopically implanted into the mammary fat pad of test mice. Bioluminescence imaging of the growth of the orthotopic 4T1-luc2 tumor was performed weekly, and representative images are shown in Fig. 1a. The level of bioluminescence intensity (BLI) and G-CSF in tumors of test mice was determined. As shown in Fig. 1b, a biphasic and relatively high level of BLI was detected in mice between day 7 and day 42 post tumor implantation (i.e., BLI >2 × 10^9^ photons/sec), in accordance with the time course pattern observed for expression of G-CSF in test mice. The population of gMDSCs expressing CD11b^+^Ly6G^+^ in white blood cells (WBCs) of peripheral blood reached 66.7% at day 7, and maintained a high level (89% to 52% of total WBCs) from day 14 to day 42 (Fig. 1c). A high level gMDSCs (≥35% of splenocytes) in the spleen of test mice was detected from day 14 to day 42 (Fig. 1c). Monocytic MDSCs (mMDSCs) expressing CD11b^+^Ly6C^+^ in WBCs of peripheral blood and in spleen tissue made up between 1% and 6% of the WBCs (Fig. 1c). In addition, the weights of tumor and spleen tissue of test mice gradually increased between day 7 to 21, but dramatically increased at day 21 post tumor implantation (Fig. 1d). The population of gMDSCs expressing CD11b^+^Ly6G^+^ in tumor masses of test mice reached 12% at day 6, and then gradually increased to 52% between day 6 and day 19. However, mMDSCs expressing CD11b^+^Ly6C^+^ in test tumor masses were maintained at an extremely low level (ca., <1% of total cells in tumor masses) from day 6 to day 19 (Fig. 1e).

### Correlation between expression levels of gMDSCs, G-CSF and rate of tumor growth and metastasis

**Figure 2:**
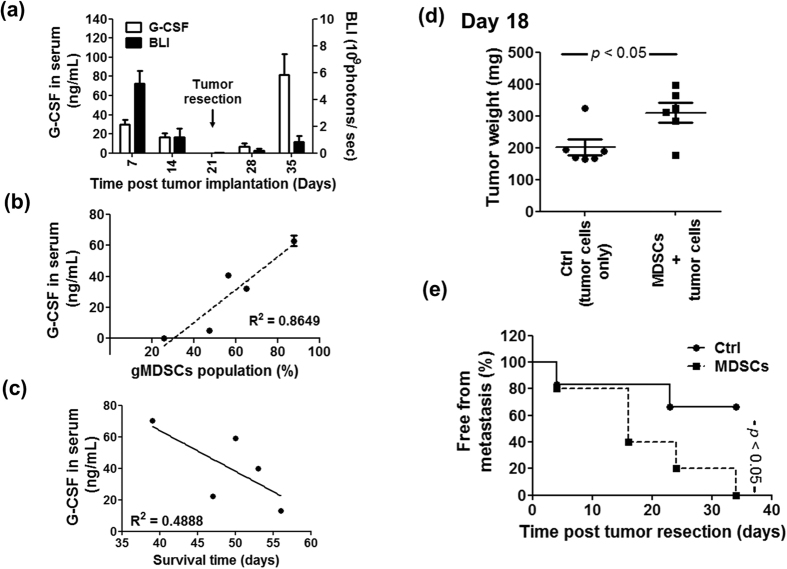
Correlation between expression levels of gMDSCs, G-CSF and the rate of tumor growth and metastasis. Test mice were orthotopically implanted with 5 × 10^5^ 4T1-luc2 cells and primary tumors were resected at day 21 post tumor implantation. (**a**) Quantitative data of bioluminescence imaging (BLI, black bar) and serum G-CSF level (white bar) in tumor-resected mice, scored between day 7 and day 35. (**b**) Correlation between population frequency of gMDSCs and serum G-CSF level in tumor-resected mice. The expression levels of gMDSCs and G-CSF in test mice were determined at day 14 post tumor resection. (**c**) Correlation between survival time (day) and serum G-CSF level. The expression data on G-CSF in test mice were determined at day 21 post tumor resection. (**d**) Test mice were co-injected orthotopically with 4T1 cells (5 × 10^5^) and granulocytic MDSCs and primary tumors were resected at day 18 post tumor implantation. Tumor mass of two test groups are shown. (**e**) The incidence of metastasis free mice treated with 4T1 only (solid circle) versus 4T1 plus MDSC (solid square) is presented.

Expression levels of gMDSCs and G-CSF have previously been shown to be closely associated with the progression of tumor growth in mouse models^27^. In order to investigate the role of gMDSCs and G-CSF in growth and metastasis of mouse mammary tumors, we orthotopically implanted 4T1-luc2 cells into the mammary fat pads of test mice. At day 21 post tumor implantation, the primary tumor mass was gently surgically removed. The level of tumor BLI and the expression of G-CSF were measured weekly, as quantified in Fig. 2a. It is interesting to note that expression level of G-CSF in serum of test mice at day 21 was dramatically reduced soon after tumor resection, indicating that the high level G-CSF detected in circulation of 4T1 tumor-bearing mice was mainly secreted by the cells of the tumor mass (Fig. 2a). After tumor resection, test mice with metastatic tumors gradually reestablished a high level expression of G-CSF in blood serum. In addition, this pattern of G-CSF expression in test mice was well correlated with the increase in the population of granulocytic MDSCs (Fig. 2b) and this increase in G-CSF level in test mice was inversely correlated with mouse survival time after tumor resection (Fig. 2b,c). These results suggest that the gMDSC population can be effectively induced by tumor-cells secreting G-CSF, and these immune cells can promote tumor growth and metastasis. We further co-injected 4T1 tumor cells (5 × 10^5^ cells) and gMDSCs (1 × 10^7^ cells) into the mammary fat pad of test mice. At 18 days post tumor implantation, the primary tumor mass was gently removed surgically. Experimental results showed that the co-transplanted gMDSCs could indeed promote tumor growth and metastasis (Fig. 2d,e). It is important to note that whereas close to 67% of the control mice were able to sustain a metastasis free state, all the gMDSC-cotreated mice were dead at 34 days post tumor resection (Fig. 2e). We therefore suggest that MDSCs and G-CSF may be useful as therapeutic targets for preventing mammary tumor growth and metastasis.

### Effect of an ethanol-extracted fraction of Bidens pilosa on the function and differentiation activities of MDSCs and on tumor metastasis

**Figure 3:**
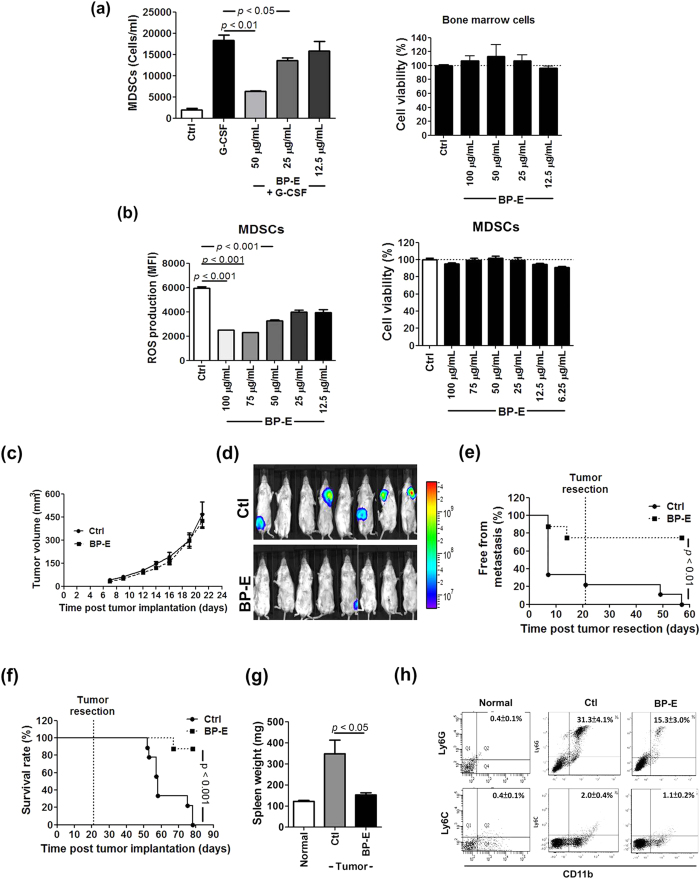
Effect of the ethanol-extracted fraction of *Bidens pilosa* on the function and differentiation of MDSCs and on tumor metastasis. (**a**) The population of granulocytic MDSCs in treated bone marrow cells was determined by flow cytometry. Cytotoxicity of an ethanol-extracted fraction of *B. pilosa* (BP-E) on bone marrow cells, revealed by MTT assay at 24 hours post treatment. (**b**) Cells were treated at serial concentrations (12.5 to 100 μg/mL) of BP-E for 24 hours and ROS expression in MDSCs was measured by incubating cells with H2DCFDA fluorescent probes. *Ex vivo* cytotoxicity of BP-E on bone marrow cells, revealed by MTT assay for 24 hours. (**c**) Tumor volume of untreated mice and mice treated with BP-E. (**d**) BLI from untreated and BP-E treated mice at 7 days post tumor resection. (**e**) The incidence of metastasis free mice in the control and BP-E treatment groups. (**f**) Survival rates of test mice. (**g**) Weight of spleen tissue in test mice on day 21 post tumor resection. (**h**) Population of granulocytic and monocytic MDSCs in spleen tissues of test mice.

To investigate potential therapeutic agents against tumor metastasis, a number of phytoextracts or the derived phytochemicals were evaluated in our laboratory for inhibition of the function and differentiation of MDSCs. The ethanol partitioned fraction of the *B. pilosa* (BP-E) plant extract significantly suppressed the G-CSF-induced differentiation of gMDSCs from bone marrow cells *ex vivo* (Fig. 3a). MTT assay showed that BP-E had no significant effect on cell viability of bone marrow cells and the derived MDSCs, at a concentration between 100 and 12.5 μg/ml (Fig. 3a). In addition, flow cytometry analysis showed that BP-E significantly inhibited the production of reactive oxygen species (ROS) in granulocytic MDSCs in a dose dependent manner (Fig. 3b). In order to evaluate a potential inhibitory effect of BP-E administered orally on tumor growth, 4T1-luc2 mouse mammary carcinoma cells were orthotopically implanted into the mammary fat pad of test mice, and subsequently examined in a tumor-resection model. At 7 days post tumor implantation, mice were divided randomly into an untreated group and a BP-E treated group (administered by oral gavage at a dose of 100 mg BP-E/kg body weight/day). BP-E had no significant effect on the growth of primary tumors, as measured by tumor volume change (Fig. 3c). We next investigated whether this oral administration of BP-E could confer an effect on tumor metastasis in a tumor resection model. For this experiment, at 21 days post orthotopic tumor implantation, the tissue mass of 4T1-luc2 tumors in test mice was surgically removed. Test animals were then randomly divided into control (untreated) and BP-E treated groups (100 mg BP-E /kg/day). BLI of the metastatic tumors of each test group at 7 days post tumor resection is shown in Fig. 3d. On this date, the incidence of metastasis in the control group was 62.5% (n = 8), whereas the incidence of metastasis in the BP-E group was only 12.5% (Fig. 3e). This is a surprisingly large difference, strongly supported by the sharp contrast in the value of BLI in Fig. 3d. It is very important to note here that, in only 7 days, a very short period of time, BP-E treatment was able to effectively suppress the tumor metastasis in this tumor resection model. It is also important to note that the tumor-resection model used is designed to mimic the conditions of human breast cancer patients following surgery. At 78 days post tumor implantation, the metastasis rate and death rate of the control group mice had reached 100%. In contrast, the metastasis rate and the death rate of BP-E treated mice were maintained at 25% and 12.5%, respectively (Fig. 3e,f). These results again strongly suggest that the early anti-metastatic effect of the treatment can be successfully maintained for a prolonged period of time. Based on the differentials seen in Fig. 3e,f, for subsequent experiments, mice were sacrificed on day 42 post tumor implantation. The results shown in Fig. 3g show that 4T1 tumor cells induced strong splenomegaly activity and BP-E dramatically reduced this tumor-induced splenomegaly (*P* < 0.05). The MDSC populations in the spleen tissue of each test group were then investigated. As shown in Fig. 3h, growth of 4T1 tumor strongly induced an accumulation of granulocytic MDSCs in the spleen and BP-E substantially reduced (with ≥50% inhibition) the tumor-induced accumulation of gMDSCs in the spleen.

### Effect of the F1 fraction of BP-E on ROS expression in MDSCs and on differentiation of MDSCs from bone marrow cells

**Figure 4:**
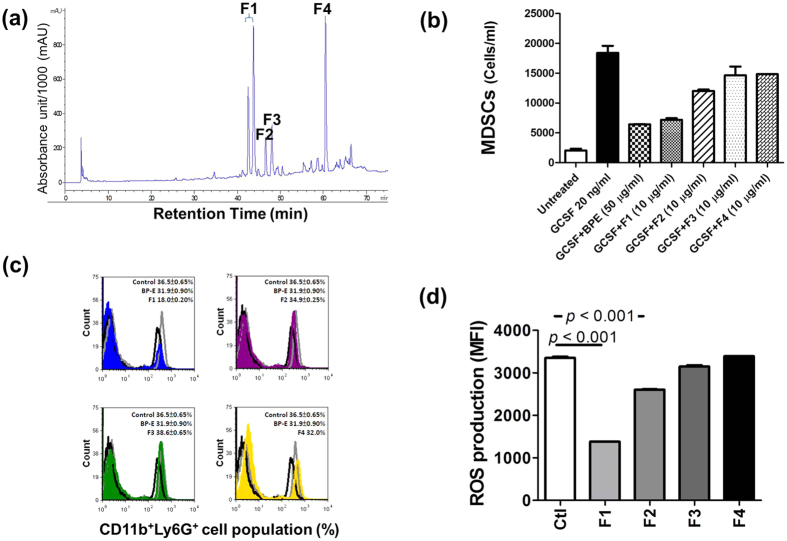
Effect of the BP-E-F1 on ROS expression in MDSCs and on differentiation of MDSCs from bone marrow cells. (**a**) HPLC profiling with an absorbance of UV 235nm of BP-E separated into 4 major sub-fractions (F1, F2, F3, and F4). (**b**) Cell number of MDSCs differentiated from bone marrow cells in treated cells was determined by flow cytometry analysis. (**c**) Population of granulocytic MDSCs (CD11b^+^Ly6G^+^) differentiated from bone marrow cells in treated mice was determined by flow cytometry analysis. (**d**) Cells were treated with four sub-fractions (F1, F2, F3, and F4) at 10 μg/mL for 24 hours and ROS expression in MDSCs was measured by incubating cells with H_2_DCFDA fluorescent probes.

To identify candidate active components or phytochemicals from the BP-E phytoextract that can confer anti-metastasis activity, BP-E was further fractioned into four sub-fractions (F1 to F4) using high performance liquid chromatography (HPLC) analysis with an absorbance of UV at 235 nm (Fig. 4a). These four subfractions were then evaluated for their inhibitory effect on the differentiation and ROS expression of MDSCs under *ex vivo* culture conditions. Figure 4b shows that BP-E as well as the derived F1 fraction (designated as BP-E-F1) significantly inhibits the G-CSF-induced differentiation of gMDSCs. Furthermore, the BP-E-F1 also strongly suppressed the ROS expression in gMDSCs (Fig. 4c,d). These results suggest that BP-E-F1 may contain the key phytochemical components of BP-E that are responsible for inhibition of the differentiation and function of MDSCs and the resultant anti-tumor metastasis activities.

### Chemical identification of F1 phytochemicals

**Figure 5:**
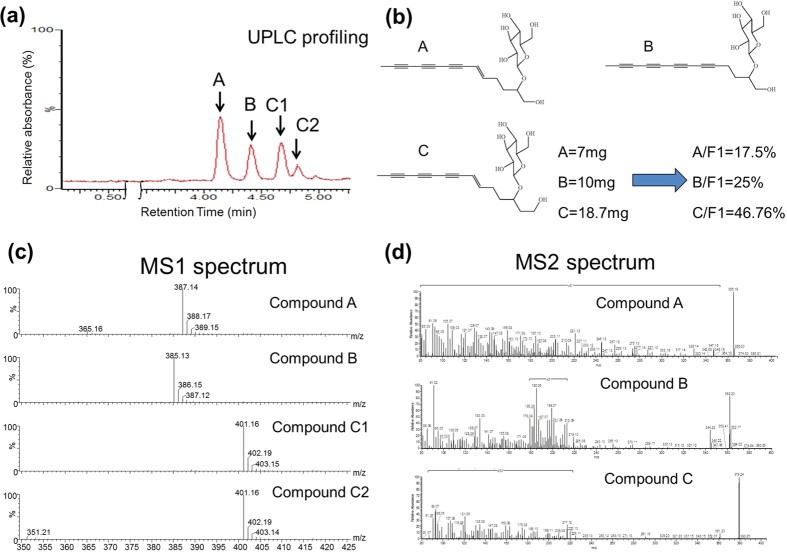
Chemical identification of F1 phytochemicals. (**a**) Chromatograph of F1 fraction by a RP-18 UPLC column. (**b**) Chemical structure of 3 major compounds (2-β-D-glucopyranosyloxy-1-hydroxy-5(E)-tridecene-7,9,11-triyne, 2-D-glucopyranosyloxy-1-hydroxytrideca-5,7,9,11-tetrayne, and 3-β-D-glucopyranosyloxy-1-hydroxy-6(E)-tetradecene-8,10,12-triyne) in F1 identified by spectroscopic methods. (**c**,**d**) Spectrum graph of 3 compounds in F1 fraction in MS/MS analysis.

Bio-organic chemical profiling of the BP-E-F1 phytochemicals was performed by using UPLC, HPLC and MS/MS assays. BP-E-F1 was initially chromatographed using a RP-18 UPLC column. Three major compounds (A-C) were isolated (Fig. 5a), and their chemical structures were subsequently elucidated by spectroscopic methods (Fig. 5b). In LC-MS-MS analysis, Compound A was identified as 2-β-D-glucopyranosyloxy-1-hydroxy-5(E)-tridecene-7,9,11-triyne by [M + H]^+^, and [M + Na]^+^ peaks were detected at m/z 365.16 and *m/z* 387.14, respectively (Fig. 5c, Peak A) and by the mass fragmentation pattern from [M + H]^+^ parent ion (Fig. 5d, Peak A). Compound B was identified as 2-D-glucopyranosyloxy-1-hydroxytrideca-5,7,9,11-tetrayne by[M + H]^+^ and [M + Na]^+^ peaks were detected at m/z 363.14 and *m/z* 385.13, respectively (Fig. 5c, Peak B), and by the mass fragmentation pattern from [M + H]^+^ parent ion (Fig. 5d, Peak B). Compound C was identified as 3-β-D-glucopyranosyloxy-1-hydroxy-6(E)-tetradecene-8,10,12-triyne by [M + H]^+^ and [M + Na]^+^ peaks detected at m/z 379.17 and *m/z* 401.16, respectively (Fig. 5c, Peak C1 and C2) and by the mass fragmentation pattern from [M + H]^+^ parent ion (Fig. 5d, Peak C1 and C2). Compounds (A-C) were also comparatively analyzed and confirmed by our previous studies^33^,^37^. The content of compounds A-C in BP-E-F1 was detected to be up to 89%.

### Effect of BP-E-F1 on tumor metastasis

**Figure 6:**
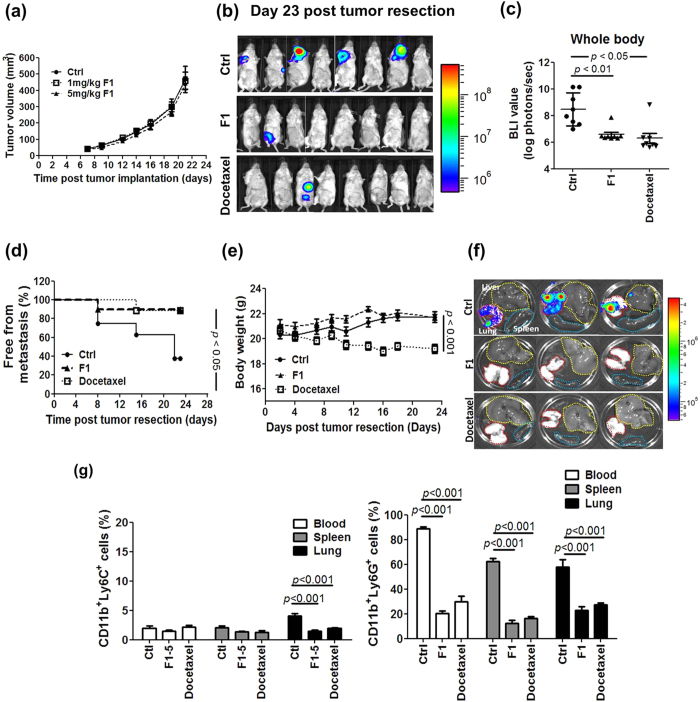
Effect of BP-E-F1 on tumor metastasis. (**a**) Tumor volume in control and BP-E-F1 group mice. (**b**) BLIs of all test mice at 23 days post tumor resection. (**c**) Quantitative data of BLIs in whole bodies of all test mice. (**d**) The incidence of metastasis free control, BP-E-F1, and docetaxol-treated mice. (**e**) Body weight of all test mice. (**f**) Representative BLI of liver, lungs, and spleen of test mice at 23 days post tumor resection. (**g**) Population of granulocytic and monocytic MDSCs in preferred organs of test mice as determined by flow cytometry.

Since we were able to separate the phytochemicals of the BP-E extract into four major fractions, we then investigated the possible inhibitory effect of the BP-E-F1 on tumor growth in our orthotopic mammary tumor growth/tumor resection mouse model. At 7 days post tumor implantation, test mice were randomly divided into untreated and BP-E-F1 groups (i.e., orally treated with 5 mg BP-E-F1/kg body weight/day). Like oral administration of BP-E, BP-E-F1 treatment had little or no significant effect on primary tumor growth, as measured by tumor volume (Fig. 6a). We next investigated the effect of BP-E-F1 on tumor metastasis in the tumor resection model. At 21 days post implantation the tumor mass was gently surgically removed. Following surgery, each treatment group was then randomly divided into control, F1 and docetaxel groups (i.e., iv injection with 10 mg docetaxel/kg every other 3 days). BLI of the metastatic tumor for each test group at 23 days post tumor resection is shown in Fig. 6b. The BLI values for each mouse were measured quantitatively and pooled for each test group. Both oral administration of BP-E-F1 and iv-injection of docetaxel were able to effectively reduce the BLI value observed for test mice with a virtually identical pattern (Fig. 6c). In addition, the metastasis rates determined for the control, F1 and DTX group mice were 62.5%, 12.5% and 12.5%, respectively, at 23 days post tumor resection (Fig. 6d). Interestingly, however, the body weights of test mice of the different treatment groups were readily distinguishable as shown in Fig. 6e. Unlike treatment with docetaxel, BP-E-F1 treatment did not result in body weight loss in test mice, and even appeared to aid weight gain. Mice were sacrificed at 23 days post tumor resection, and the lungs, liver and spleen of test mice were excised and tumor metastasis was measured by BLI. Figure 6f shows that the lung is the preferred organ for metastasis of 4T1 tumor cells in test mice, and treatment with BP-E-F1 and docetaxel effectively inhibited tumor metastasis into the lung. In addition, treatment with BP-E-F1 or docetaxel significantly reduced the 4T1 tumor-induced accumulation of granulocytic MDSCs in the lung, peripheral blood and spleen of test mice (Fig. 6g).

### BP-E-F1 inhibits the effect of MDSC activities on tumor growth and metastasis

**Figure 7:**
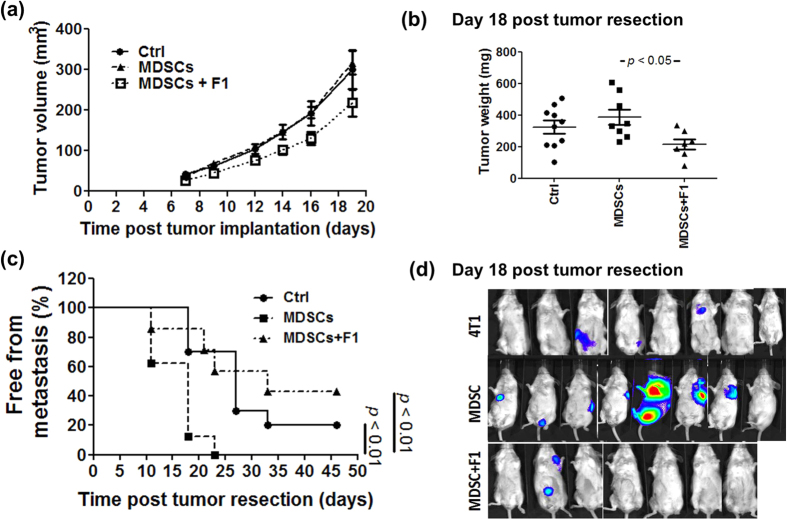
BP-E-F1 inhibits effects of MDSCs on tumor growth and metastasis. (**a**) Tumor volume of control, BP-E-F1, and BP-E-F1 + MDSC group mice. (**b**) Tumor weight of al test groups at 18 days post tumor implantation. (**c**) The metastasis free mice in all test groups. (**d**) BLIs of all test groups at 14 days post tumor resection.

Our current results suggest that BP-E as well as its derived F1 fraction can effectively suppress tumor metastasis via inhibition of the differentiation of MDSCs from bone marrow cells and the accumulation of MDSCs in the tumor microenvironment. For subsequent experiments, we injected 4T1 cells alone or co-injected with granulocytic MDSCs into the mammary fat pad of test mice. Starting at 7 days post tumor implantation, mice were fed orally with F1 (5 mg/kg) every day. At 18 days post tumor implantation, the tumor masses of test mice were gently removed surgically and measured. F1 treatment significantly inhibited the effect of MDSCs on tumor growth as measured weekly by tumor volume and tumor mass (Fig. 7a,b). In addition, F1 treatment significantly suppressed MDSC-promoted tumor metastasis after tumor resection (Fig. 7c,d). Taken together, our results suggest that MDSC activity plays a key role in 4T1 tumor metastasis and could serve as a therapeutic target for fighting tumor growth and metastasis. BP-E as well as BP-E-F1 can suppress 4T1 tumor metastasis via an inhibition of the differentiation of MDSCs from bone marrow cells and the accumulation of MDSCs in specific tumor microenvironments.

## Discussion

In our current study, we established a luminescent murine mammary 4T1-luc2 orthotopic tumor model, with which tumor resection and subsequent monitoring of tumor metastasis were conducted. We systemically investigated the role of MDSCs in tumor growth and metastasis in this system. Our findings suggest that an immunotherapeutic strategy that involves MDSCs may be usefully employed to treat against metastatic cancers. Our results showed that granulocytic MDSCs (gMDSCs) are the major MDSC population accumulated in the peripheral blood and spleen tissue of 4T1 tumor-bearing mice, and are present from the early stage to the later stage of tumor growth (Fig. 1c). In addition, we also observed that the percentage of gMDSCs at the tumor site at day 21 post tumor implantation were upregulated to 27% (data not shown), and 4T1 tumor cells express consistently high levels of G-CSF, resulting in the induction of abundant gMDSCs in test mice. The tumor site and spleen tissues are considered to be the key reservoirs of MDSCs and their precursor cells^38^,^39^. Due to the massive accumulation of gMDSCs, spleen and tumor site tissues of tumor-bearing mice became dramatically and rapidly swollen, as seen clearly at 21 days post tumor implantation (Fig. 1d). We propose that this massive gMDSC accumulation, may effectively hijack the host immune system, and render it ineffective at inducing efficacious antitumor immunities. The role of gMDSCs in promoting tumor growth and metastasis was further confirmed by co-implantation of tumor cells with gMDSCs into the mammary fat pad of test mice. Co-implantation of tumor cells with gMDSCs resulted in increased tumor weight and a higher incidence of metastasis compared with implantation of tumor cells alone (Fig. 2d,e). Together these results and the results of previous findings from other studies^40^,^41^, strongly suggest that MDSCs can play a determinant or check point role in the programming of tumor-induced immunosuppression, facilitating tumor growth and metastasis against host immunity. Therefore, instead of directly targeting and killing tumor cells, effective control and/or suppression of MDSC production may be employed as a front line strategy for immunotherapy against metastatic tumors in specified cancer patients expressing high level MDSCs in body fluid tissues^42^,^43^,^44^.

A growing body of evidence suggests that chemotherapy, performed as a systemic therapy for metastatic cancers, often does not benefit cancer patients, and instead may often impair host immunities, resulting in the promotion of tumor growth and spread^45^,^46^,^47^. Our results demonstrate that oral administration of an ethanol extract of *B. pilosa* (BP-E) or the phytochemicals from a derived fraction (BP-E-F1) can significantly suppress 4T1 mammary metastasis. Importantly, the efficacy of the F1 fraction in inhibition of metastasis and MDSC accumulation was at a level comparable with docetaxel treatment (Fig. 6). Furthermore, mice fed BP-E-F1 phytochemicals, composed mainly of three specific polyacetylenes, showed better general health than docetaxel-treated mice, and treatment with the polyacetylene phytochemicals, unlike docetaxel, did not result in body weight loss (Fig. 6e) or hair loss in the tested mice. A direct comparison of the efficacy, ease of drug delivery, cytotoxicity and other side effects of BP-E-F1 and the current standard clinical drug docetaxel, suggests that BP-E, or the polyacetylenes derived from BP-E-F1 may have high potential for clinical application as an anti-cancer metastasis agent for use in combination with existing chemotherapy drugs.

For pharmacological application, bioavailability is defined as the fraction of the administered dose of a test drug that reaches a systemic level in blood circulation. We initially determined the absolute bioavailability of the three major polyacetylenic glycoside compounds (A, B, C) of BP-E-F1 in the blood of test mice. Oral administration was also used to investigate the effect of BP-E-F1 on suppression of 4T1 metastasis. The bioavailability of the three BP-E-F1 compounds (A-C) was assessed in BALB/c mice (n = 12) via administration of BP-E-F1 by either intravenous (iv) or oral delivery, both at 10 mg/kg. As seen in Supplementary Fig. S1a, the area under curve (AUC) for oral administration and iv administration in the bioavailability study were experimentally obtained at 282.8 and 1268 mg.min/l, respectively. The absolute bioavailability level for oral administration can hence be calculated to be 22.3%. We next determined the presence or absence, and the concentration of the three compounds (A-C) in bone, kidney, liver, lung and spleen tissues after the administration of BP-E-F1 delivered via oral administration. Organs of test mice (n = 3) were collected at 2 hours post oral administration of BP-E-F1. The concentration of the three compounds (A-C) in different organs was detected (Supplementary Fig. S1b). Compounds (A-C) of BP-E-F1 were maintained at a relatively high concentration in serum, kidney, bone, liver, lung and spleen tissues of BALB/c mice at 20 min to 2 h post oral delivery, indicating that the active phytocompounds of BP-E-F1 can be readily and directly absorbed into blood circulation and target organs, effecting the suppression of development and function of gMDSCs. Promisingly, these BP-E-F1 phytochemicals are more readily bioavailable via oral administration.

Results of Fig. 3 to Fig. 5 show that BP-E-F1 significantly suppressed the differentiation of MDSCs from bone marrow cells and the functionality of MDSCs, *in vitro* and *in vivo*. Tumor-derived G-CSF has been demonstrated to play a key role in promotion of gMDSC development^27^. In order to investigate the potential mechanistic role of BP-E-F1 in inhibition of gMDSC differentiation, we evaluated the effect of intravenous administration of recombinant G-CSF, aiming to elicit gMDSC activities. Exogenously supplemented recombinant G-CSF significantly promoted the increase of granulocyte percentage in the peripheral blood of test mice from 16.1% (level in untreated mice) to 49.1% (Supplementary Fig. S2a). This activity apparently stimulated the phosphorylation of STAT3, a key transcription factor for differentiation and function of MDSCs in test bone marrow cells *in vivo* (Supplementary Fig. S2b). Furthermore, oral feeding with F1 partially suppressed the percentage of granulocytes in the peripheral blood of treated mice (Supplementary Fig. S2a) and effectively (up to 63%) reduced the phosphorylation level of STAT3 in bone marrow cells of G-CSF-treated mice (Supplementary Fig. S2b). In an *ex vivo* experiment, BP-E and F1 treatments also significantly reduced the phosphorylation of STAT3 in gMDSCs (Supplementary Fig. S2c). Collectively, our results suggest that BP-E and BP-E-F1 can effectively suppress the differentiation and function of gMDSCs via inhibition of tumor-derived G-CSF-induced activation of STAT3. Together, the results from our present study lead us to suggest that a group of specific plant polyacetylenic glycosides (BP-E-F1) can mediate potent anti-metastasis activity via targeting defined granulocytic MDSC functions. Our findings point to the possibility of the development of a novel class of immunotherapeutic agents for use against metastatic cancers.

## Methods

### Extraction of plant tissues, phytochemical isolation and identification

*Bidens pilosa* Linn var. radiata (Asteraceae) plants were grown on farms in Sanxia District, New Taipei City, Taiwan, in 2013. Air-dried shoot, leaf and root tissues of whole plants, weighing 228.2 g were imbibed, stirred and extracted in 2.28 liters of 95% ethanol (EtOH) at room temperature for three days. This total crude extract was evaporated in a vacuum to yield a dried residue (6.3955 g) that was then resuspended in methanol (MeOH) and eluted with a water-MeOH mixture of decreasing polarity, using a PR-18 preparative HPLC column (Cosmosil C18, 4.6 mm × 250 mm) with a flow rate of 0.5 ml/min, and detected at UV 235 nm to give a total of four sub-fractions (BP-E-F1 − BP-E-F4). The BP-E-F1 (eluent of 73.5% MeOH/water from the PR-18 column) was identified as the bioactive fraction. Therefore, BP-E-F1 was repeatedly separated by the same eluent with 70% to 72% MeOH in water for further use in *in vitro* and *in vivo* studies.

BP-E-F1 was subsequently separated via chromatography by a RP-18 UPLC column (Acquity UPLC HSS C-18 column 2.1 × 150 mm, 1.8 um), eluted with 30% to 32% acetonitrile (ACN) with 0.2% formic acid (FA), to give a total of three 2^nd^ sub-fractions (designated as BP-E-F1-A, -B, and –C (C_1_ and C_2_)). These 2^nd^ sub-fractions were then further separated from BP-E-F1 (40 mg) by the same PR-18 preparative HPLC column as mentioned above and eluted with 31.2% ACN with 0.05% FA to yield compounds A (BP-E-F1-A, 7 mg), B (BP-E-F1-B, 10 mg), and C (BP-E-F1-C1 + BP-E-F1-C2, 18.79 mg). LC-MS/MS was performed to identify the chemical formulas of compounds A, B and C. The LC-ESI-Orbitrap Elite MS data were processed using the Xcalibur version 2.7.0 SP1 (Thermo Scientific). For the LC-MS analysis, data were generated by intensities of the peaks, with the retention time (RT) and the *m/z* data pairs were used to identify each peak.

### Construction of 4T1-luc2 cells

293T cells were transfected with pMD.G, pCMVΔR8.91, and pIF4g.As2.luc.bla to construct a lentivirus vector expressing luc2 gene. Twenty-four hours later, cell culture media were collected and employed to transfect 4T1 cells with the constructed viral vector. Blasticidin S (10 μg/mL) was applied to select the single clones of transfected 4T1-luc2 cells. Transgenic 4T1-luc2 cells were cultured and maintained in RPMI-1640 medium supplemented with 10 μg/mL blasticidin S, 10% fetal bovine serum, 1 mM penicillin/streptomycin and 1 mM sodium pyruvate in an incubator at 37 °C in 5% CO_2_ and 95% humidity.

### Mouse tumor studies

Mouse mammary carcinoma 4T1-luc2 cells (5 × 10^5^ cells/100 μl PBS)^48^ were orthotopically implanted into the mammary fat-pad of BALB/c mice. To evaluate the effect of BP-E or BP-E-F1 on anti-tumor growth activities, mice were divided into untreated group and BP-E treated group (orally treated with 100 mg BP-E/kg body weight/day) or BP-E-F1 treated group (orally treated with 5 mg BP-E-F1/kg body weight/day) at 7 days post tumor cell implantation. Primary tumor growth was evaluated by measuring tumor weight every other day and by monitoring the bioluminescence imaging of mammary tumors every 7 days. For the tumor resection model, at day 21 post tumor implantation, the tumor mass (about 0.3 g in general) was gently removed surgically, and the wounds were sutured back. To evaluate the effect of BP-E or BP-E-F1 on anti-metastasis activity, mice were then administered with control (PBS) and BP-E (orally treated with 100 mg/kg body weight/day) or BP-E-F1 (orally treated with 5 mg /kg body weight/day) or docetaxol (10 mg/kg body weight) by intravenous injection (2 injections/week) post tumor resection. Bioluminescence imaging of metastatic tumors was then followed and monitored using a non-invasive *in vivo* imaging system (IVIS). All experiments were performed in accordance with relevant guidelines, and experimental protocols involving animals were approved by the Institutional Animal Care and Utilization Committee (IACUC) of Academia Sinica, Taipei (IACUC ID: 10-12-105).

### Cell population analysis

Lung tissues of test mice were harvested and minced with 150 U/mL type I collagenase in tissue grinders 20 to 50 times. After digestion and lysing with ACK buffer, ground tissues were collected and filtered through a 40 μm cell strainer. Spleen tissues were minced gently with PBS in tissue grinders. After lysing with ACK buffer, cells were collected for further analysis. Blood samples were lysed with ACK buffer 3 times and harvested for further analysis. Test cells were collected and stained with anti-CD11b, and anti-Ly6G/Ly-6C for flow cytometry analysis.

### Isolation of gMDSCs

To purify Ly-6G^+^ MDSCs, splenocytes of tumor-bearing mice were harvested and erythrocytes were depleted by ACK buffer. Then, splenocytes were incubated with anti–Ly-6G-biotin Abs for 20 minutes followed by positive selection using anti-biotin microbeads following the manufacturer’s instructions (Miltenyi Biotec).

### Bone marrow cell preparation

BALB/c mouse bone marrow cells from femora and tibiae were depleted of RBCs with ACK lysis buffer and cultured in RPMI 1640 medium supplemented with 20 ng/ml GM-CSF, 10% fetal bovine serum, 50 μM 2-mercaptoethanol, 100 unit/ml penicillin and 100 μg/ml streptomycin in a humidified 5% CO_2_ incubator at 37 °C^49^.

### Immunoblotting

Cell lysates were prepared using M-PER Mammalian Protein Extraction Reagent [5 mM bicine buffer, 4-(2-aminoethyl)benzenesulfonyl fluoride (AEBSF 0.3 mM), leupeptin (10 μg/ml) and aprotonin (2 μg/ml)]. Lysates were run on 5% to 20% gradient polyacrylamide-sodium dodecyl sulphate (SDS) gels (20 μg protein per lane), proteins were then transferred onto Hybond-ECL membranes (GE-Healthcare, Amersham, UK) and immunoblotted with anti-G-CSFR antibody, anti-stat3 antibody, or anti-phosphorylated stat3 antibody. Protein bands were detected by enhanced chemiluminescence (Clarity Western ECL Substrate, BioRad) and developed by autoradiography.

### Detection of serum G-CSF by ELISA

Serum from test mice and conditional medium of test cell cultures were collected and stored at −80 °C until use. Samples were checked for expression levels of G-CSF (R&D Systems) and quantified at a wavelength of 450 nm using a Biotek PowerWave HT spectrophotometer.

### Antibodies

Anti-stat3 antibody and anti-phosphorylated stat3 antibody were purchased from Cell Signaling Technology. Anti-G-CSFR antibody was purchased from Abcam.

### Statistical analysis

Data are presented as fold-changes or percentages with mean ± SEM as indicated in the corresponding figure legends. All statistical analyses were determined using GraphPad software. For comparison between multiple data sets, a one-way ANOVA analysis with Tukey–Kramer method was performed.

## Additional Information

**How to cite this article**: Wei, W.-C. *et al*. Inhibiting MDSC differentiation from bone marrow with phytochemical polyacetylenes drastically impairs tumor metastasis. *Sci. Rep.* **6**, 36663; doi: 10.1038/srep36663 (2016).

**Publisher’s note:** Springer Nature remains neutral with regard to jurisdictional claims in published maps and institutional affiliations.

## Supplementary Material

Supplementary Information
