# Supplementary material for: Inhibiting MDSC differentiation from bone marrow with phytochemical polyacetylenes drastically impairs tumor metastasis
Source: Sci Rep. 2016 Nov 18;6:36663. doi: 10.1038/srep36663 (PMC5114612; doi:10.1038/srep36663)
Supplement: Supplementary Information [file srep36663-s1.pdf]

Inhibiting MDSC differentiation from bone marrow with phytochemical polyacetylenes drastically impairs tumor metastasis

Wen-Chi Wei[1]†, Sheng-Yen Lin[1,2]†, Chun-Wen Lan[1], Yu-Chen Huang[1], Chih-Yu Lin[1], Pei-Wen Hsiao[1], Yet-Ran Chen[1] , Wen-Chin Yang[1], and Ning-Sun Yang[1*]

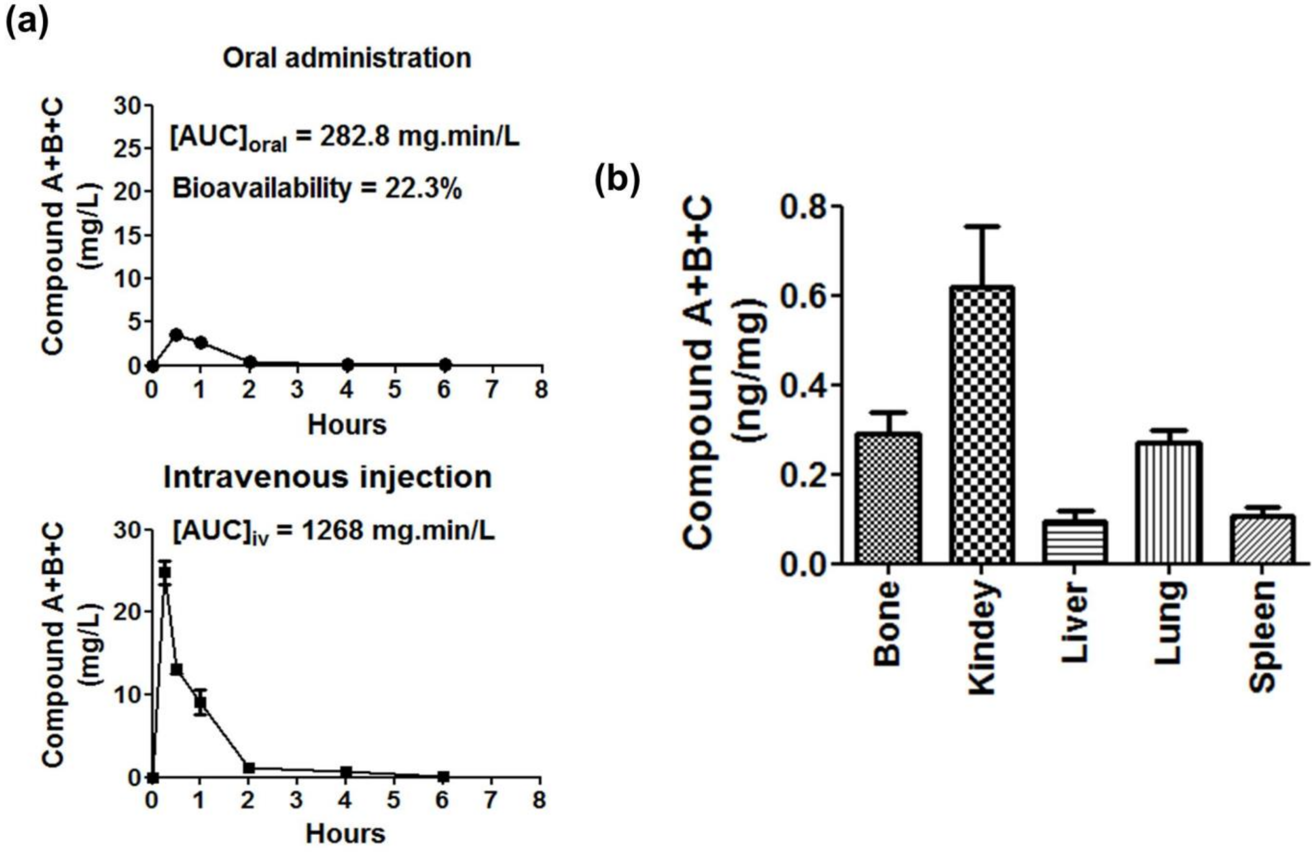

Supplemental Fig. 1. Pharmacokinetic study of BP-E-F1.

(a) The concentrations of the three compounds (A-C) of BP-E-F1 in test sera were determined by liquid chromatography-tandem mass spectrometry (LC/MS/MS). The absolute bioavailability of oral administration was then determined by the dose-corrected area under the curve (AUC) of oral administration divided by AUC of iv administration. (b) The bone, kidney, lung, liver and spleen tissues in BP-E-F1 treated mice were collected and the concentrations of the three compounds (A, B, and C) were detected by liquid chromatography-tandem mass spectrometry (LC-MS/MS).

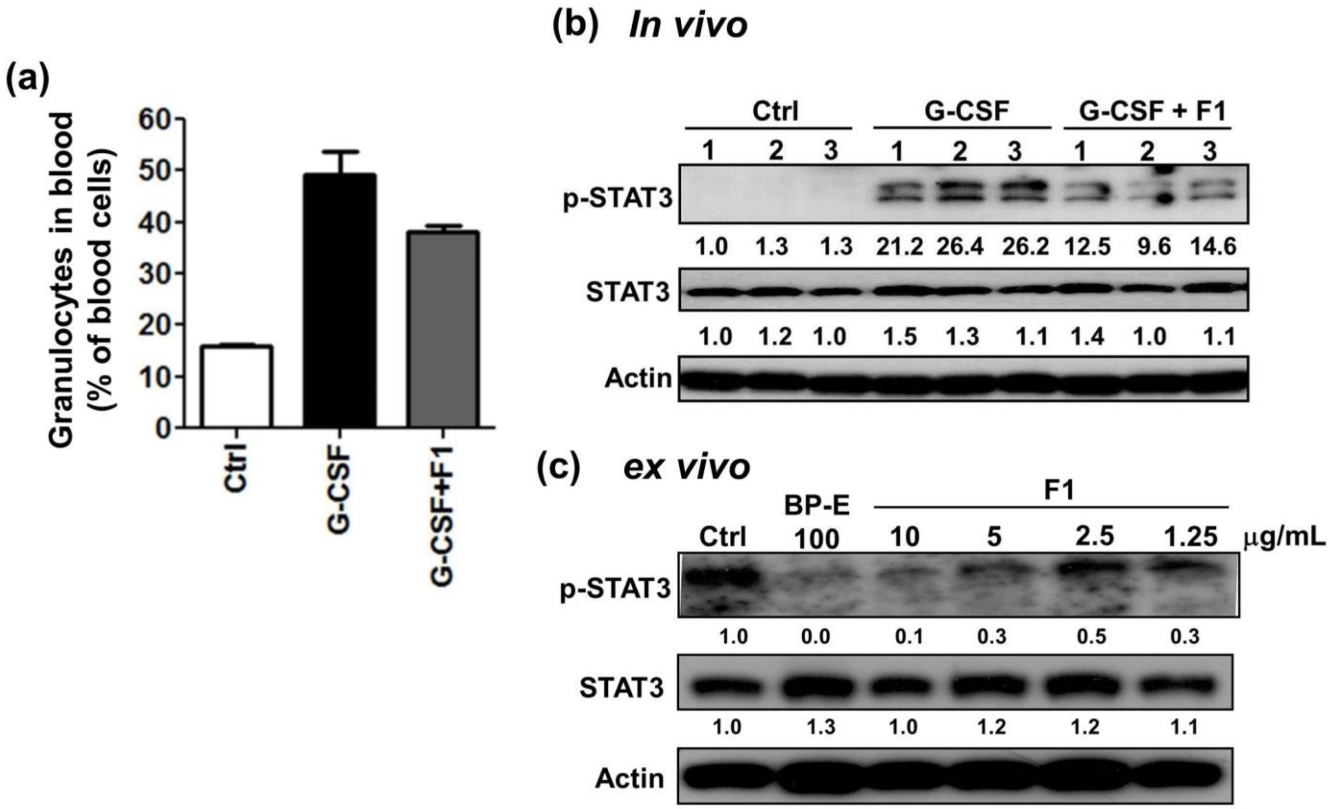

Supplemental Fig. 2. BP-E-F1 inhibits G-CSF-induced granulocyte differentiation and signaling transduction.

(a) Cell number of granulocytes in the peripheral blood of test mice was determined by using a hematology analyzer. (b) Expression of phosphorylation of STAT3 and total STAT3 in representative bone marrow cells *in vivo* were measured by western blot analysis. The histochemical staining of p-STAT3 shown as tracks #1 and 2 in the G-CSF+F1 treatment groups were apparently "contaminated or nonspecifically stained" by three black dots, and these stainings were not scanned intentionally to avoid misleading activity intensities. (c) Expression of phosphorylation of STAT3 and total STAT3 in treated gMDSCs *ex vivo* were determined by western blot analysis.
